# Supplementary material for: Sugary interfaces mitigate contact damage where stiff meets soft
Source: Nat Commun. 2016 Jun 16;7:11923. doi: 10.1038/ncomms11923 (PMC4912638; doi:10.1038/ncomms11923)
Supplement: Supplementary Information — Supplementary Figures 1-4, Supplementary Tables 1-6, Supplementary Discussion and Supplementary References [file ncomms11923-s1.pdf]

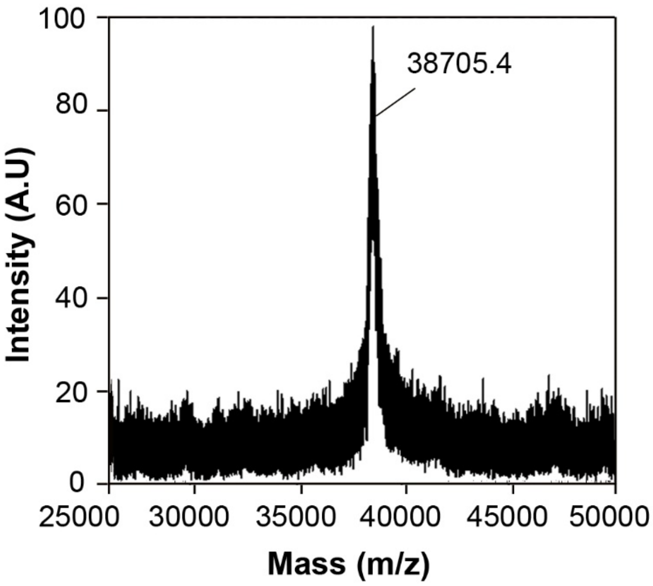

Supplementary Figure 1. MALDI-TOF mass spectrometry of apfp-1 purified by HPLC.

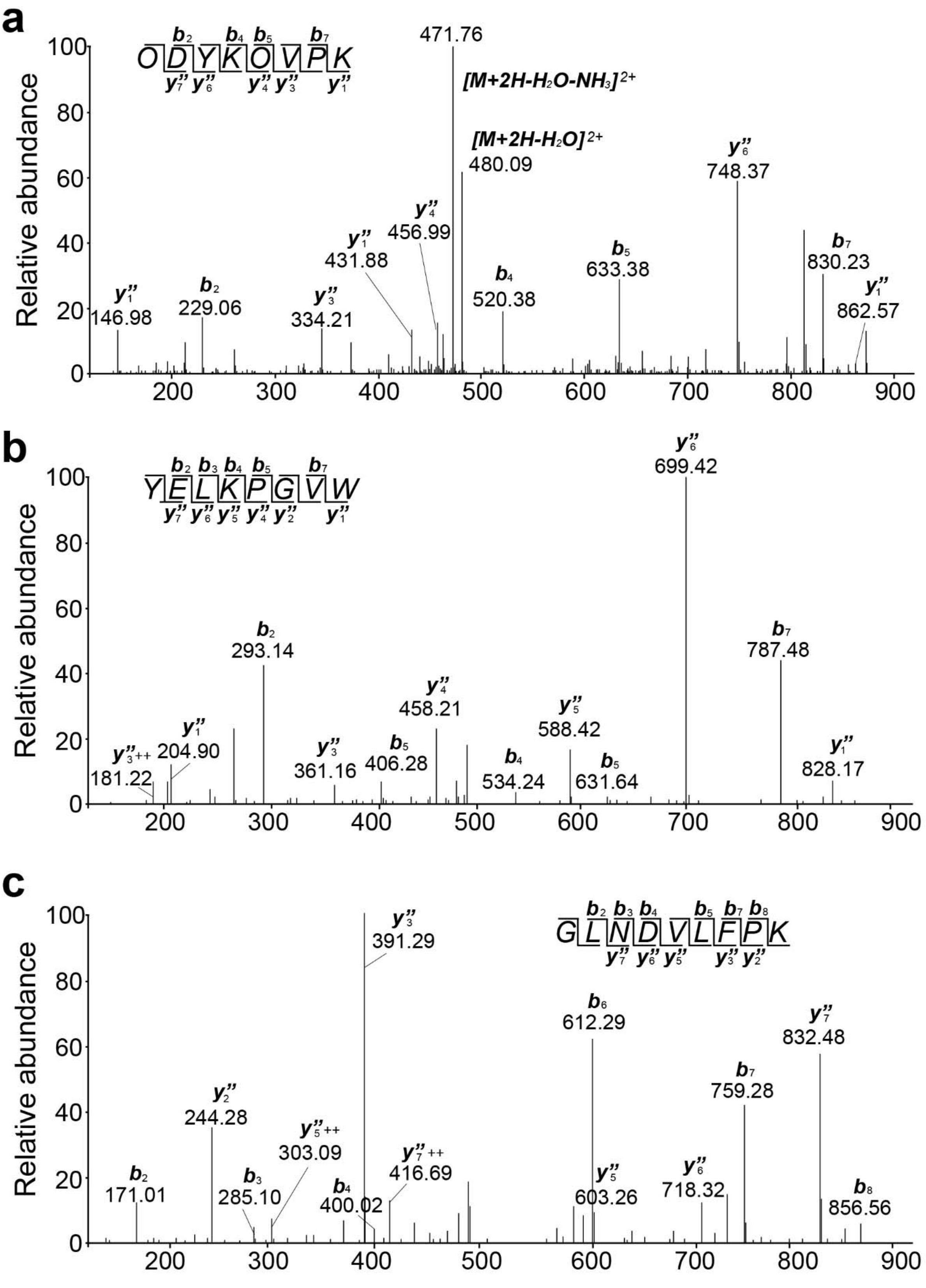

Supplementary Figure 2. Trypic peptide from *Atrina* DOPA-containing protein identified by ESI-MS/MS. "O" denotes 4-trans hydroxyproline.

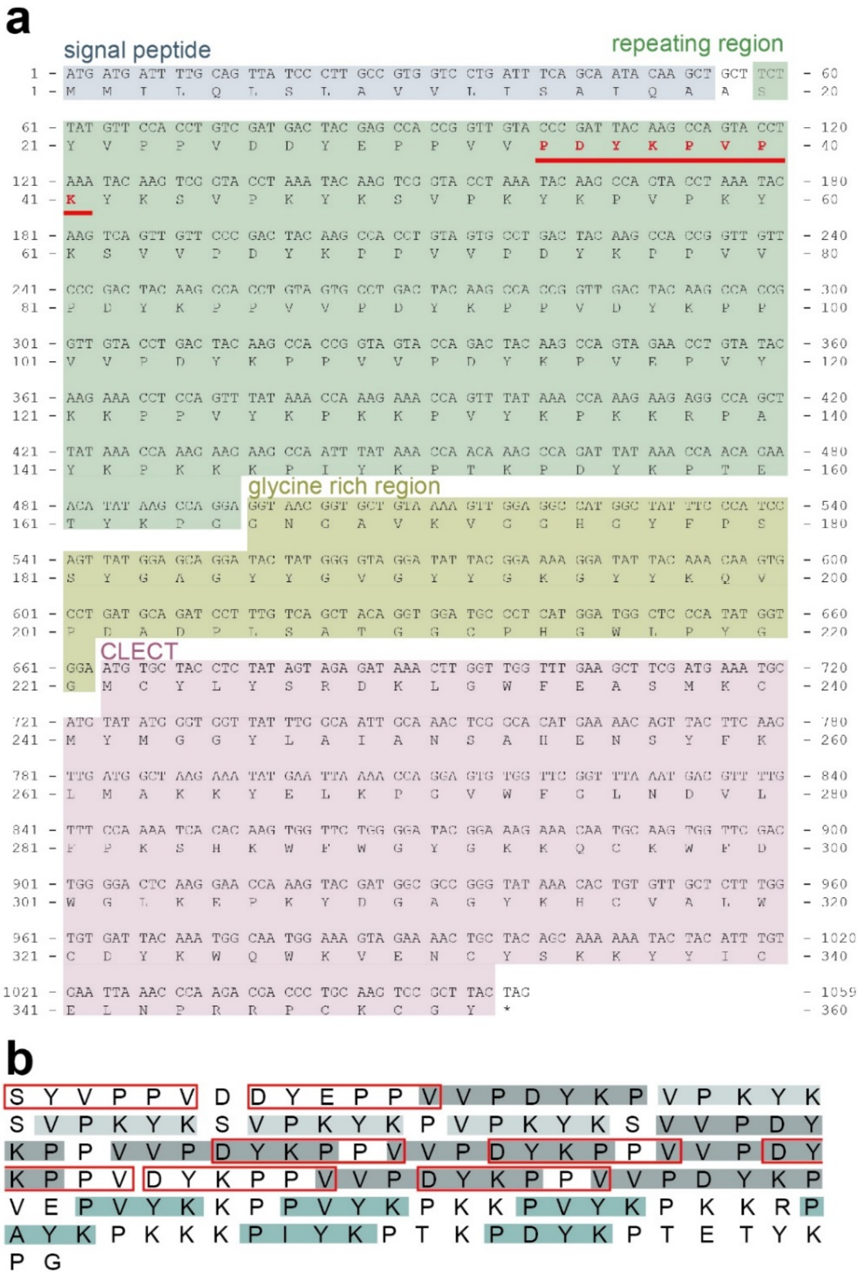

Supplementary Figure 3. The nucleotide sequence, amino acid sequences, and regions in apfp-1. (a) Nucleotide and amino acid sequences of cloned apfp-1. Signal peptide is highlighted in blue, Glycine-rich region is highlighted in yellow, CLECT domain is highlighted in purple and the peptides isolated following ESI-MS/MS are underlined in red. The nucleotide sequence for apfp-1 gene has been deposited in the GenBank database under GenBank Accession Number KF951620. **(b)** The apfp-1 sequence between signal peptide and glycine rich region is the repeating region, which is highlighted in green. The grey rectangle represents VVPDYKP repeat, the sky blue rectangle represents the VPKYK repeat, the blue rectangle represents the P_YK repeat and the red rectangle represents the S/DY_PPV repeat.

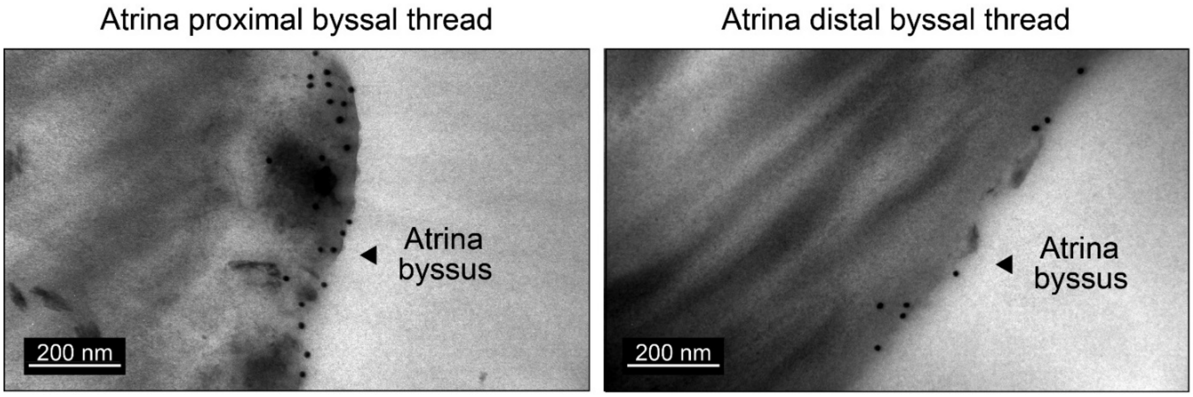

Supplementary Figure 4. Immuno-gold labeling of apfp-1 in cryo-TEM sections through *Atrina* byssal threads. Thin sections samples were labeled with anti-apfp-1 antibody, followed by gold conjugated secondary antibodies.

Supplementary Table 1. ***Atrina pectinata*** **byssal thread and soft tissue (adductor muscle) material properties. Values given means ± standard error. N ≥ 25.**

Conditions	Young's Modulus (MPa)	Extensibility (mm/mm)	Tensile Strength (MPa)
Soft tissue	0.1555±0.051	0.778±0.247	0.127±0.054
Byssus	268.12±45.89	0.301±0.137	22.61±13.49
Byssus (EDTA)	134.47±23.02	0.334±0.007	13.15±7.41

Supplementary Table 2. Nano indentation measurement of ***Atrina*** **byssus.**

	Cuticle	Core	Epoxy
Hardness (GPa)	0.22±0.02<br>n = 8	0.22±0.05<br>n = 7	0.12±0.007<br>n = 2

Supplementary Table 3. Amino acid composition of ***Atrina pectinata*****,** ***Pinna nobilis*** **and** ***Mytilus*** **byssus, purified apfp-1 and predicted apfp-1.**

Amino Acids	Byssus *Atrina pectinata*	Byssus *Pinna nobilis*	Byssus *Mytilus*	*Atrina* Purified Protein Amino Acid Composition	*Atrina* cDNA Deduced Amino Acid Composition
HyPro	-	-	4.1	-	-
Asx	9.7±0.1	10.3	8.5	7.6±0.0	7.2
Thr	4.7±0.1	4.1	4.2	1.7±0.3	1.2
Ser	8.6±0.1	7.1	6.2	3.8±0.2	3.9
Glx	5.9±0.6	6.5	8.6	4.7±0.6	3.6
Pro	11.4±0.3	11.4	6.6	15.9±1.7	15.9

Gly	9.6±0.1	6.9	18.2	9.2±1.0	8.4
Ala	4.9±0.0	2.7	9.9	5.3±0.3	3.9
Cys	1.8±0.5	3.0	1.2	0.1±0.1	3.0
Val	8.9±0.1	7.1	1.0	9.8±0.5	9.9
Met	1.7±0.0	1.9	1.2	1.3±0.2	1.5
Ile	4.1±0.1	3.3	2.5	1.1±0.1	0.9
Leu	4.9±0.0	5.0	1.3	4.1±0.3	3.6
Tyr	2.8±0.1	6.5	2.3	5.8±0.2	13.2
DOPA	0.5±0.0	10.1	13.2	5.2±0.5	0.0
Phe	2.2±0.0	2.8	2.6	2.3±0.1	2.1
His	1.8±0.4	2.3	3.0	2.8±0.3	1.5
Lys	10.8±0.2	12.4	5.5	17.5±0.6	16.2
Arg	5.6±0.1	6.3	6.7	1.8±0.2	1.2
Trp	0.8±0.2	-	-	-	3.0

Supplementary Table 4. Biochemical analysis of *Atrina* byssus. The experiment is conducted five times and the average value with standard deviation (SD) is presented.

Component	Percentage ± SD (wt %)
Protein	99.46 ± 0.09
Lipids	0.16 ± 0.07
Carbohydrate	0.38 ± 0.03

Supplementary Table 5. Analysis of metal content in *Atrina* byssus using ICP-MS. The experiment is conducted three times and the average value with standard deviation (SD) is presented.

Analyte	Average Concentration ± SD (wt %)
Ca	0.53 ± 0.21
Fe	0.42 ± 0.04
Zn	0.08 ± 0.01

Supplementary Table 6. Consensus repeat sequences in fp1 proteins from several mussels.

Species	Consensus	Repeats	Reference
Atrina pectinate	VVPDYKP	(7)	Present
Atrina pectinate	VPKYK_	(4)	Present
Atrina pectinate	PVYK_	(6)	Present
Perna canaliculus	PYVK_	(72)	Zhao, 2005[1]
Aulacomya ater	AGYGGVK_		Burzio, 2000[2]
Trichomya hirsute	SYYPK_		Rzepecki, 1991[3]
Modiolus modiolus	SSYYPK_		Rzepecki, 1991[3]
Choromytilus choros	AKPSYPTGYKPPVK_		Burzio. 2000[2]
Mytilus edulis	AKPSYPPTYK_____	(71)	Filpula, 1990[4]
Mytilus galloprovincialis	AKPSYPPTYK_____	(85)	Inoue, 1994[5]

SUPPLEMENTARY DISCUSSION

Tensile test of byssus threads. The present study analyzed biomechanical properties of *Atrina* byssal threads and surrounding soft tissue (adductor muscle) under wet condition, with and without the addition of 50 mM EDTA. The results for the biomechanical analysis of the byssus threads and the soft tissue are shown in Supplementary Table 1. In the case of Young's modulus and tensile strength of the byssus was more than 200 fold higher than those of the soft tissue.

Biochemical and element analysis of byssus threads. The amino acid composition of *Atrina* byssus was overall similar to that of byssi from the previously studied *Pinna nobilis*[6] and other *Atrina* species (Supplementary Table 3). Serine (~9 mol %), glycine (~9 mol %), valine (~9 mol %), proline (~11 mol %), and lysine (~15 mol%) are the dominant amino acids in *Atrina* byssus. The other notable amino acid constituent was DOPA (~2 mol%), a key molecule for mussel underwater adhesion. Notwithstanding this similarity with *Mytilus* byssus, there is a significant amino acid bias between the two compositions (Supplementary Table 1). The most abundant amino acid of *Mytilus* byssus is glycine (~18 mol %) whereas in *Atrina*, glycine amounts to only ~9 mol % of the total residues. On the other hand, while *Mytilus* byssus contains only traces of valine (~1 mol %), in *Atrina,* valine abundance is nine times higher at ~9 mol %. In addition to valine, *Atrina* threads contain higher percentages of proline (~11 mol %), isoleucine (~4 mol %), leucine (~5 mol %) and lysine (~11 mol %) compared to *Mytilus* byssus (~6 mol %, 2 mol %, 1 mol %, and 5 mol %, respectively).The freshly harvested *Atrina pectinata* has a lustrous gold color. The byssus is largely organic, and biochemical analysis of individual threads showed a composition of 99.46 ± 0.09 wt % protein, 0.16 ± 0.07 wt % lipids and 0.38 ± 0.03 wt % carbohydrate (see Supplementary Table 4). The overall composition of *Atrina* byssus was similar to that of *Mytilus* byssus, which contains 95.0±3.0 wt% proteins and 2.5 wt % hexoses.[7]

Using ICP as described in *Methods,* we detected several key metal content in the byssus. The three main metal atoms detected in the byssus are shown in Supplementary Table 5. The analysis of the byssus threads revealed a metal content of 0.53±0.12 % wt for Ca, 0.42 ± 0.02 % wt for Fe and 0.08±0.00% wt for Zn.

Extraction and purification of *Atrina pectinata* foot protein 1 (apfp-1). *Atrina* foot proteins were isolated by a prior protocol based on mussel foot extraction.[7] Soluble proteins from a 5% acetic acid *Atrina* foot extract were separated by gel permeation chromatography and the collected fractions examined by AU-PAGE. During C-8 reverse phase HPLC, the peak was present at ~ 48% acetonitrile. Given the persistent co-elution, a second GPC analysis, this time performed at a flow rate of 0.2 ml min^{-1}, was done in an effort to better separate the proteins. AU-PAGE of the second GPC suggested a successful separation of single proteins. In order to confirm that the proteins isolated from the HPLC peak is apfp-1, SDS-PAGE was performed and the apparent molecular weights of the band was determined as ~38 kDa.

Characterization of apfp-1. Peak HPLC fractions were also subjected to MALDI-TOF mass spectrometry. MALDI-TOF verified that the purified proteins have similar masses to the cDNA deduced apfp-1 sequence. As shown in Fig. S1, the observed masses by MALDI-TOF of the purified pools were, 38705.4, which is consistent with the apparent mass from SDS-PAGE (Fig.1.e). The mass difference between apfp-1 by MALDI-TOF mass spectrometry and the cDNA deduced apfp-1 sequence without the signal peptide was around ~220 Da, suggesting apfp-1 has around 13~14 DOPA residues if all the post-translational modification corresponds to DOPA.

Peptide sequencing. The peptides were subjected to ESI-MS/MS peptide sequencing after in situ trypsin digestion. Three peptides were identified following collision induced dissociation by ESI-MS/MS: ODYKOVPK (in which “O” denotes hydroxyproline) YELKPGVW, and GLNDVLFPK (see Fig. S2). In order to obtain a partial cDNA deduced sequence of the *Atrina* foot protein, 3’ RACE was performed using degenerate oligonucleotides designed from the known amino acid sequence of the tryptic peptide PDYKPVPK (codons for proline in all cases of detected hydroxyproline) and the Universal Primer Mix from Clontech. After cloning and sequencing the partial apfp-1 cDNA, a specific primer was designed to amplify the 5’ end of the gene and obtain the full length cDNA. The apfp-1 full-length cDNA has a length of 1071 bp (corresponding to 360 amino acids).

Sequence identification. The complete cDNA-deduced protein sequence was subjected to *in silico* analyses by various “Tools” in Swiss-Prot. apfp-1 has a signal peptide sequence 18

residue long, and cleavage between A-18 and A-19 is predicted by “SIGNALP” (ExPASy). Following the signal peptide, after 13 amino acids, the protein contains ~10 somewhat degenerate repeats of the octapeptide VVPDYKPP. Then, subsequent to the first octapeptide repeat are four insertions of the VPKYKS/P hexapeptide sequence. The fifth and sixth heptapeptide repeats are separated by the PVDYKPP sequence. After the heptapeptide repeats, the sequence PVYK is repeated six times, with valine replaced by alanine, isoleucine and aspartic acid for the fourth, fifth and sixth repeat, respectively. The P(V/A/I/D)YK repeats are punctuated mostly by proline and lysine residues.

The C-terminus of the protein is predicted by the conserved domain search on NCBI to consist of a Ca-dependent or C-type lectin module (CLECT domain) (Fig. S3A). ProtParam program (ExPASy) predicts a molecular weight of 38485.8 Da and a theoretical pI of 9.47 for the deduced amino acid sequence without the signal peptide. The predicted molecular weights for the protein without the CLECT and the CLECT domain by itself are 22678.3 and 15825.5 Da, respectively.

Detection of apfp-1 using antibody. *Atrina* byssal threads were analyzed for the presence of apfp-1 using a polyclonal antibody raised against the peptide VLFPKSHKWFWGYGKKQCKWFD, that is part of the CLECT domain. In the proximal part of *Atrina* byssus, the antibody localizes the protein around the matrix that surrounds each byssal thread. In the distal part, less antibody labelling is visible, labelling being present mostly around the bundle of fibers.

Supplementary References

1 Zhao, H. & Waite, J.H. Coating proteins: structure and cross-Linking in fp-1 from the green shell mussel *Perna canaliculus*. *Biochemistry* **44**, 15915-15923 (2005).

2 Burzio, L.A., Saéz, C., Pardo, J., Waite, J.H. & Burzio, L.O. The adhesive protein of *Choromytilus chorus* (Molina, 1782) and *Aulacomya ater* (Molina, 1782): a proline-rich and a glycine-rich polyphenolic protein. *BBA-Protein Struct. M* **1479**, 315-320 (2000).

3 Rzepecki, L.M., Chin, S.S., Waite, J.H. & Lavin, M.F. Molecular diversity of marine glues: polyphenolic proteins from five mussel species. *Mol Mar Biol Biotechnol* **1**, 78-88 (1991).

4 Filpula, D.R., Lee, S.M., Link, R.P., Strausberg, S.L. & Strausberg, R.L. Structural and functional repetition in a marine mussel adhesive protein. *Biotechnol. Prog.* **6**, 171-177 (1990).

5 Inoue, K. & Odo, S. The adhesive protein cDNA of *Mytilus galloprovincialis* encodes decapeptide repeats but no hexapeptide motif. *Biol. Bull.* **186**, 349-355 (1994).

6 Pujol, J.P., Rolland, M., Larsy, S. & Vinet, S. Comparative study of the amino acid composition of the byssus in some common bivalve molluscs. *Comp. Biochem. Phy.* **34**, 193-201 (1970).

7 Marsh, J. & Weinstein, D. Simple charring method for determination of lipids. *J. Lipid Res.* **7**, 574 (1966).
